# Recent decadal enhancement of Meiyu–Baiu heavy rainfall over East Asia

**DOI:** 10.1038/s41598-021-93006-0

**Published:** 2021-07-07

**Authors:** Hiroshi G. Takahashi, Hatsuki Fujinami

**Affiliations:** 1 Department of Geography, Tokyo Metropolitan University, Hachioji, Japan; 2 Institute for Space–Earth Environmental Research, Nagoya University, Nagoya, Japan

**Keywords:** Climate sciences, Natural hazards

## Abstract

East of Eurasia, moist air is transported poleward, forming the Meiyu–Baiu front over East Asia in late June and early July. Recently, unusually heavy rainfall may have increased, causing catastrophic flooding in East Asia. Here, unique 23-year precipitation satellite radar data confirm recent enhancement in Meiyu–Baiu heavy rainfall from eastern China to southwestern Japan, which is also evident from independent conventional observations. Decadal changes in rainfall have been physically consistent with enhanced transport of water vapour due to the intensified Pacific subtropical high associated with weakened tropical cyclone activity over the Northwest Pacific. Furthermore, the upper-tropospheric trough, associated with wave train along the subtropical jet, influenced Meiyu–Baiu precipitation over East Asia. Long-term and continuous satellite radar observations reveal that the frequency of heavy precipitation along the Meiyu–Baiu front has increased in the last 22 years. In particular, heavy precipitation (10 mm/h) increased by 24% between 1998–2008 and 2009–2019, and the abruptly-changed level likely induced recent meteorological disasters across East Asia. This trend may also explain the severity of the 2020 Meiyu–Baiu season. Over the last decade, this front has likely transitioned to a new climate state, which requires adaptation of disaster prevention approaches.

## Introduction

**Figure 1:**
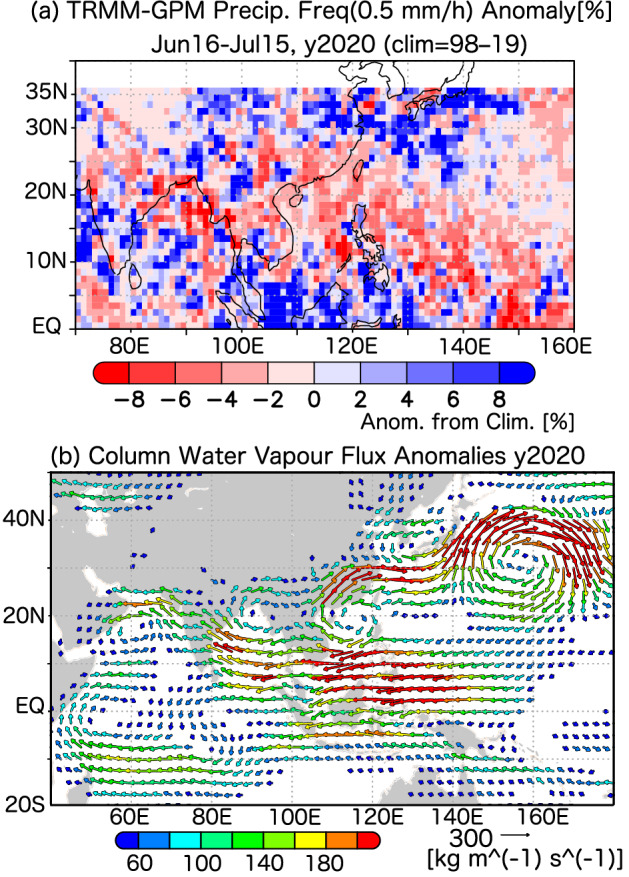
Maps of (**a**) precipitation and (**b**) vertically-integrated water vapour flux anomalies in the 2020 Meiyu–Baiu season, as compared to the mean climate, defined as the 22-year mean for 1998–2019 for all meteorological variables. The Meiyu–Baiu season was assumed to occur from 16 June to 15 July every year (for plotting, see Methods). In (**b**), water vapour fluxes smaller than 40 kg/m/s were omitted from the plotted data. Figure generated with GrADS 2.2.1 (http://cola.gmu.edu/grads).

The frequency of meteorological disasters^1^ has recently increased, including the unusually heavy rainfall events and floods that occurred across East Asia in 2020 (Fig. 1), which were associated with a rainy season of the East Asian monsoon known as the Meiyu–Baiu (or simply Meiyu or Baiu) rainfall season^2–5^. The Meiyu–Baiu front is brought by the confluence of moist, poleward, low-level jet streams originating from the low-level cyclonic flow of the Asian monsoon and anticyclonic flow along the rim of the western North Pacific subtropical high (WNPSH). These jet streams are the major pathways by which moisture is transported from the tropics to the mid-latitudes.

The characteristics of the East Asian monsoon and Meiyu–Baiu rainfall have been examined in many studies^6,7^. The development of this system is associated with the stepwise sub-seasonal progression of the East Asian monsoon^8,9^. In particular, the Meiyu–Baiu system and the WNPSH are associated with the development of the western North Pacific monsoon^10^, and the rainfall activity over the western North Pacific is associated with the development of tropical cyclones (TCs) along a monsoon trough^11^. This trough expands zonally across the northern Bay of Bengal, the Indochina Peninsula, and the South China Sea to the Philippine Sea. In the context of global climate change, TC activity along this trough is key to explaining Asian monsoon precipitation in the summer, which also affects the development of the WNPSH^12^.

Long-term and decadal variabilities in Meiyu–Baiu rainfall are crucial issues affecting climatic variability over the Asian monsoon region. However, recent trends in such rainfall have not been evident from observations, though numerical experiments have been conducted to explore future changes in Meiyu–Baiu rainfall activity^13–19^. Recently, a 23-year satellite precipitation radar dataset from the Tropical Rainfall Measuring Mission–Global Precipitation Measurement Mission (TRMM–GPM) has become available. The satellite precipitation radar observations provide accurate rainfall data via the direct estimation of precipitation, including heavy rainfall. Before satellite precipitation radar observations, rainfall was primarily estimated based on rain gauge measurements and cloud activities derived from infrared imagers. However, cloud activity and precipitation do not record the same phenomena.

In this study, we examined recent decadal trends in Meiyu–Baiu rainfall over the last two decades using satellite precipitation radar data. We focused on the active Meiyu–Baiu season, from the second half of June to the first half of July, when severe meteorological disasters frequently occur across the East Asian monsoon region. Along with the recent decadal changes in Meiyu–Baiu rainfall, we explored the variability of atmospheric circulation, such as the transport of water vapour and upper-tropospheric circulations.

## Results

**Figure 2:**
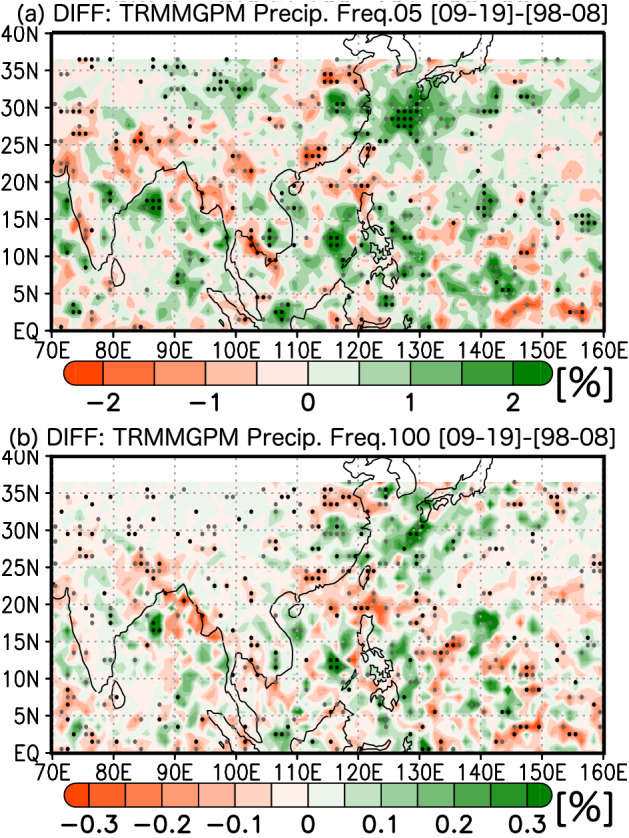
Twenty-two-year changes in frequency of Meiyu–Baiu rainfall (i.e. the differences between the two 11-year periods), with statistical significance shown by black and grey dots, which denote 95% and 90% confidence intervals, respectively. Precipitation was counted when the rainfall rate exceeded the threshold value ((**a**)**:** 0.5 mm/h, (**b**): 10 mm/h). For definition and plotting, see Methods. Figure generated with GrADS 2.2.1 (http://cola.gmu.edu/grads).

To understand the possibility of recent decadal enhancement in Meiyu–Baiu precipitation, Fig. 2 shows the recent decadal changes in the rainfall frequency observed by satellite precipitation radars. Rainfall is frequently observed on the western coasts of tropical Asian monsoon regions, in the western North Pacific, and in the Meiyu–Baiu region (Supplementary Fig. 1a,b) from the second half of June to the first half of July (i.e. the Meiyu–Baiu season). Meiyu–Baiu rainfall activity is oriented SW–NE over East Asia. As a key systematic signal of the 22-year changes in precipitation frequency, the rainfall frequency (> 0.5 mm/h) has increased along the zonal band of 30° N from 115° E to 135° E, with some meridional extension (Fig. 2a), indicating the decadal enhancement of Meiyu–Baiu rainfall activity. Other minor organised signals were observed over a coastal region in South China and over the northern South China Sea. Including these regions, decreased precipitation was found over East China near 35° N, as well as in parts of the western North Pacific, which have been drying. The dipole precipitation trends over the Meiyu–Baiu front and the western North Pacific are similar to the Pacific–Japan teleconnection^20^.

Long-term satellite precipitation radar observations (see Methods) allowed us to retrieve 5-km (horizontal scale) torrential precipitation events over the tropics and subtropics. It is difficult to accurately determine the strength of precipitation from most cloud-based precipitation estimates. For example, high precipitation height does not always result in heavy rainfall^21^. Additionally, radar networks installed on Earth’s surface can only cover nearby areas.

We explored the possible recent enhancement of the Meiyu–Baiu front for heavy rainfall (> 10 mm/h). The frequency of heavy rainfall events has increased (Fig. 2b), which may have directly led to the recent meteorological disasters across East Asia. We also found a similar tendency when we used other threshold values (1.0, 2.0, and 20.0 mm/h; Supplementary Figs. 2, 3, 4). Thus, the recent enhancement of the Meiyu–Baiu precipitation was due to both heavy and moderate precipitation events increasing in frequency. The robustness of these signals was evaluated from different aspects, including cloud activity, which is independent of satellite precipitation data. Long before the TRMM–GPM era, outgoing long-wave radiation (OLR) was continuously observed (since the 1970s) to understand cloud activity. OLR observations have many advantages (see Methods); however, clouds cannot be used to accurately distinguish between heavy or moderate rainfall events.

Long-term changes in the convective index (CI) showed a similar tendency to the TRMM–GPM results, suggesting that Meiyu–Baiu rainfall has been enhanced since the end of the 1990s and into the 2010s (Supplementary Fig. 5). The number of active convective days has significantly increased around the Meiyu–Baiu front from eastern China to southwestern Japan. Additionally, convective activity has been suppressed over the region from southern coastal China to the Philippines. These decadal drying tendencies can be observed over a zonal band across North India and the Indochina Peninsula to the Philippines and correspond to a monsoon trough with TC activity and abundant rainfall^22^. Decadal drying signals along the monsoon trough can also be observed from the TRMM–GPM data (Fig. 2a, b).

**Figure 3:**
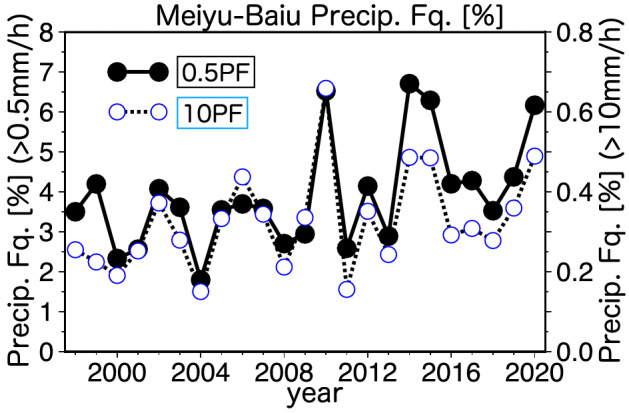
Time series of interannual variations in the frequency of Meiyu–Baiu band precipitation (27–33º N, 115–135º E). Precipitation (0.5 mm/h; black solid line with closed circles) and heavy precipitation (10.0 mm/h; dashed line with opened circles) frequencies from 1998–2020 are shown. The definition of rainfall frequency is the same as in Fig. 2. The rainfall frequency was averaged along the Meiyu–Baiu band in the Meiyu–Baiu season each year.

The decadal variability in Meiyu–Baiu precipitation was examined using the time series of precipitation frequency. The TRMM–GPM-based time series of moderate and heavy precipitation (Fig. 3) clearly showed recent decadal enhancement in precipitation, along with increased interannual variability. In particular, extremely high values were observed in the wet years of the 2010s. The precipitation in 2020 was also very high, which indicates that this trend has continued at least through the Meiyu–Baiu season of 2020 (Fig. 1). We also confirmed the decadal enhancement of Meiyu–Baiu rainfall from the CI. The CI time series based on cloud activity was remarkably similar to that of the moderate rainfall frequency, indicating that the radar-derived enhancement in precipitation was robust (Supplementary Fig. 6). Differences between the radar and OLR observations are discussed in the “Discussion and Conclusions” section. The interannual correlation between the area-averaged (27–33° N, 115–135° E,) time series of rainfall frequency (0.5 mm/h) and the CI was high (r = 0.84).

**Figure 4:**
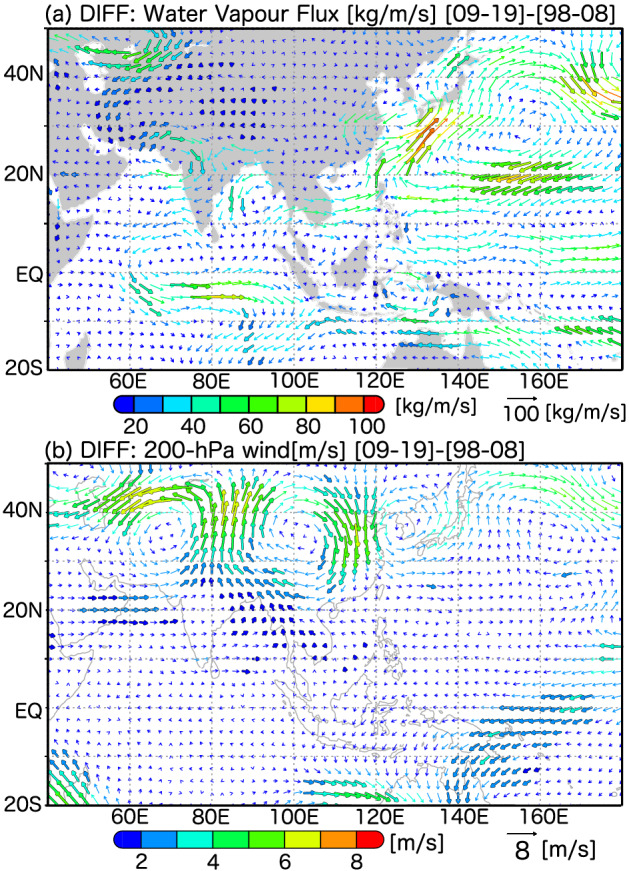
(**a**) Trends (22-year changes; differences between the two 11-year periods) in vertically-integrated water vapour fluxes from Earth’s surface to the top of the atmosphere during the Meiyu–Baiu season and (**b**) trends in 200-hPa winds. The statistical significances at a 95% confidence interval are shown by the black lines drawn around the edges of the vectors. Figure generated with GrADS 2.2.1 (http://cola.gmu.edu/grads).

Along with the decadal changes in Meiyu–Baiu rainfall, we examined the long-term changes in vertically-integrated water vapour fluxes and upper-tropospheric circulations during the Meiyu–Baiu seasons from 1998 to 2019 (Fig. 4). The difference in the former revealed that the transport of water vapour along the rim of the WNPSH from the South China Sea and Philippine Sea to the East China Sea has been enhanced. Anomalous cyclonic circulations were found over the East China Sea centred at ~ 32° N, 130° E, which enhanced the convergence of water vapour and Meiyu–Baiu rainfall along the frontal band. Additionally, anomalous anticyclonic circulations formed over the Philippine Sea, centred at ~ 20° N, 130° E, and blocked the transport of water vapour to the Philippine Sea, thereby reinforcing such transport to the Meiyu–Baiu front by redirecting the moist monsoonal flow northward. This anticyclonic anomaly over the Philippine Sea was considered to be the westward expansion of the WNPSH.

Anticyclonic signals may be associated with suppressed convective rainfall due to inactive TC activity (Supplementary Fig. 7). Low-level atmospheric circulations displayed the same tendencies as fluxes in water vapour (Supplementary Fig. 8), and a similar enhancement in water vapour transport was observed during the 2020 Meiyu–Baiu season. However, the transport of water vapour was enhanced over a more western path along the western edge of the WNPSH (Fig. 1b). These results suggest that TCs were inactive along the monsoon trough during the 2020 Meiyu–Baiu season (Supplementary Fig. 9). The enhancement of the WNPSH is associated with warm sea surface temperatures over the Indian Ocean on an interannual time-scale^23–27^, and this was also the case in 2020^28,29^. Thus, it is quite possible that this interannual variability of the WNPSH is partly associated with the decadal enhancement of the WNPSH. However, further investigation is needed to ascertain if it can explain the decadal enhancement of the WNPSH in the same mechanism.

Long-term changes in upper-tropospheric circulations were noted around the Meiyu–Baiu rainfall band (Fig. 4b). Over and around the Korean Peninsula, anomalous cyclonic circulations were found, which can be explained by a sharp enhancement of an upper-tropospheric trough or cold vortex. In front of the upper-tropospheric trough, synoptic-scale upward motion was induced, which is consistent with enhanced Meiyu–Baiu rainfall activity over East Asia^30–34^. Moreover, the upper-tropospheric trough corresponded to cyclonic anomalies in the lower-troposphere, and upper-tropospheric wave signals were observed along the subtropical jet from east of the Tibetan Plateau to the North Pacific Ocean, implying that the mid-latitudinal upper-tropospheric waves were clearly associated with a recent decadal enhancement in Meiyu–Baiu rainfall. Compared with the first half of our study period (1998 to 2009), this wave pattern likely began to appear more frequently in the second half. Additionally, 200-hPa wind anomalies in 2020 showed similar trends to the decadal changes in upper-tropospheric circulation (Supplementary Fig. 10), the latter of which were very similar across East Asia and along the subtropical jet.

We examined the decrease in rainfall frequency over the western North Pacific, focusing on the monsoon trough, where TCs are climatologically dominant. To understand the anomalous anticyclonic circulation, we analysed the activity of TCs, including weaker TCs, based on the perturbation kinetic energy (PKE; see Methods) because both stronger and weaker TCs can bring much rainfall. A decadal weakening of TC activity in the last 22 years was detected over the Philippine Sea and the South China Sea along the monsoon trough (Fig. 1b). These changes were consistent with the decadal decreases in rainfall and westward expansion of the WNPSH over the monsoon trough (Fig. 2 and Supplementary Fig. 8), which was also associated with Meiyu–Baiu rainfall through the decadal enhancement of water vapour fluxes. In the 2020 season, we found clear westward expansion of the WNPSH (Supplementary Fig. 11) and suppressed TC activity along the monsoon trough (Supplementary Fig. 9), which were also consistent with the dry anomaly (Fig. 1a). These results imply that the decadal weakening of the TC activity along the monsoon trough has enhanced the Meiyu–Baiu rainfall activity. The decadal enhancement in the Meiyu–Baiu front from eastern China to the southern coast of Japan was also apparent in the decadal enhancement in PKE (Supplementary Figs. 7 and 9), which is associated with large-scale and meso-scale convective systems along the Meiyu–Baiu front.

## Discussion and conclusions

Meiyu–Baiu rainfall activity was likely enhanced in the 2010s, based on the unique long-term satellite datasets analysed in this study (see “Methods” section). Although radar precipitation observations are very accurate, sampling is infrequent. Therefore, to fully utilise these accurate but infrequent observations, the accumulation of long-term data and statistical analyses were necessary. To further increase the robustness of the results of precipitation radar analyses, we examined decadal changes from multiple independent data sources, and the decadal enhancement of Meiyu–Baiu rainfall activity was also confirmed from conventional but stable OLR observations. Decadal changes in atmospheric circulations also support the reported enhancement in Meiyu–Baiu rainfall. This suggests that recent and unusually severe meteorological disasters across East Asia may be attributable to the recent enhancement of Meiyu–Baiu precipitation activity. It is notable that this tendency can also explain the Meiyu–Baiu activity observed in 2020, which induced torrential and prolonged rainfall and severe flooding in China and Japan. Thus, the recent enhancement of Meiyu–Baiu activity is correlated with and likely the cause of the unusually catastrophic Meiyu–Baiu season in 2020.

The decadal enhancement of Meiyu–Baiu rainfall is associated with both tropical and mid-latitudinal influences. Along with the westward expansion of the WNPSH due to weakened TC activity, more water vapour is transported to the Meiyu–Baiu front. Simultaneously, enhanced cyclonic circulation over the East China Sea also intensifies the transport of water vapour, which may be coupled with upper-tropospheric wave activity. Although, we could not quantify the contribution of each component, it is likely that this situation has continued into 2020. Detailed quantification of the contributions of each component and their long-term changes are needed in the future.

In addition to the last 22 years, for which precipitation radar data are available, we assessed the long-term changes in convective activity over the past 42 years (Supplementary Fig. 6). We found that another decadal peak in Meiyu–Baiu rainfall occurred in the 1990s. However, this activity was lower than that observed in the 2010s. Both the mean Meiyu–Baiu activity and its interannual variability in the 2010s were much greater than in the 2000s. Comparing the time series of the radar-derived and OLR-derived Meiyu–Baiu activity, we observed extremely high values only in the precipitation radar data in the wet years of the 2010s. Thus, the comparison (Supplementary Fig. 6) suggests that cloud activity does not capture the full extent of heavy rainfall.

To explain these decadal changes, the wetter conditions should be considered^35^ because the atmospheric circulation of the East Asian summer monsoon has been weakened^36,37^. Although extreme meteorological events can be explained by changes in atmospheric circulation, our results suggest that the recent activity of the Meiyu–Baiu front has likely changed to a new climate state, which may be due to wetter and warmer thermodynamic conditions. Actually, precipitation associated with the Meiyu–Baiu front can intensify in warmer conditions^38^. While the exact mechanism of the observed decadal enhancement is unclear, the evidence clearly reveals that such enhancement has occurred in the last 22 years.

## Methods

Data from the Tropical Rainfall Measuring Mission–Global Precipitation Measurement Mission (TRMM, December 1997 to December 2013; GPM, January 2014 to December 2020) and the unified precipitation dataset (TRMGPM-V6) were used as the satellite precipitation measurements in this study. We only used the precipitation radar products due to the precipitation accuracy, though there are many TRMM–GPM products. The key advantage of the TRMM–GPM product used in this study is the accuracy of the precipitation estimates derived from the TRMM and GPM satellite radars, which can directly estimate precipitation much more accurately than other sensors, such as passive microwave and infrared imagers. However, the satellites record observations infrequently due to their narrow swaths. Thus, a long period of time is required to determine the overall climate of a region and its long-term changes^39,40^. With the use of long-term and accurate observational data over 23 years, we analysed the possible enhancement of Meiyu–Baiu heavy rainfall for the first time, which may have induced recent meteorological disasters in East Asia.

To obtain robust results from the long-term satellite precipitation radar datasets, we calculated the rainfall frequencies with several threshold values: 0.5, 1.0, 2.0, 10.0, and 20.0 mm/h. When the rainfall intensity of an observation exceeded a threshold value, we qualified the precipitation within a range of rainfall intensity. The rainfall frequency, expressed as a percentage, was defined by dividing the number of the observed surface precipitation pixels by the total number of observation pixels. Because the GPM Dual-frequency Precipitation Radar (DPR) is more sensitive than the TRMM Precipitation Radar (PR), it can detect weaker precipitation events. However, top priority was given to long-term consistency for climate analyses. Eventually, to understand which ranges of rainfall intensity increased, the decadal changes in rainfall frequency using several threshold values were investigated. For plotting, the rainfall frequency was compiled on a 1° × 1° grid. No data is shown by not plotting basically north of 36° N (Fig. 1a, 2 a and b, Supplementary Figs. 1, 2, 3, 4). During the TRMM-era (1998–2013), only tropical and subtropical regions (approximately 36° S–36° N) were observed due to the satellite orbit of the TRMM.

To examine decadal changes based on frequent observations of cloud activity, which are independent of TRMM–GPM observations, we also used outgoing long-wave radiation (OLR) data from 1979–2020. The advantages of OLR observations include a longer and more continuous time series when compared with TRMM–GPM data and a higher observation frequency, which can reduce sampling biases. In many previous studies, cloud activity has been silhouetted against precipitation activity over the Asian monsoon regions, including the Meiyu–Baiu front. Thus, due to the much higher sampling of OLR, we could derive a smoother spatial pattern for Meiyu–Baiu convective activity. However, because OLR data are quite different from those obtained by precipitation radars, OLR observations cannot be used to accurately distinguish between heavy and moderate rainfall. To estimate the Meiyu–Baiu activity from clouds, we employed a traditional convective index (CI). We defined vigorous cloud activity as having an OLR value of < 220 W/m^2^, which indicates a well-developed cloud, and then counted the frequency of the CI (OLR < 220 W/m^2^) during the climatologically mature Meiyu–Baiu period (16 June to 15 July) in each year.

To understand the transport of water vapour and atmospheric circulations related to Meiyu–Baiu activity, we analysed water vapour fields using the Japanese 55-year reanalysis (JRA-55) dataset^41^, and the vertically-integrated water vapour flux from Earth’s surface to the top of the atmosphere. We also analysed horizontal winds in the lower and upper troposphere. To understand tropical cyclone (TC) activity, including that of weak TCs, we calculated the perturbation kinetic energy (PKE = *u′*^2^ + *v′*^2^) at 850-hPa, using a disturbance component of the low-level zonal (meridional) wind, *u*’ (*v*’), which was the deviation from the 11-day running mean of *u* (*v*).

Decadal changes were simply defined as the difference between the two 11-year periods from 1998–2008 and 2009–2019 to make maximum use of the TRMM–GPM datasets. We confirmed that nearly the same results could be obtained when two 10-year periods from 2000–2009 and 2010–2019 were selected instead. We statistically analysed the precipitation frequency and CI at confidence intervals of 95% and 90% (two-sided Student’s *t*-test). To analyse atmospheric circulations, we only considered values that were statistically significant at the 95% level, while for the 850-hPa PKE condition, only values that were significant at the 90% level were considered.

## Supplementary Information

Supplementary Information.

## Data Availability

Data supporting the findings of this study are publicly available from the following links: satellite precipitation radar (https://www.eorc.jaxa.jp/GPM/en/archives.html) through free registration, OLR (https://psl.noaa.gov/data/gridded/data.interp_OLR.html) for free, and JRA-55 (https://jra.kishou.go.jp/JRA-55/) through free registration.
