# Supplementary material for: Recent decadal enhancement of Meiyu–Baiu heavy rainfall over East Asia
Source: Sci Rep. 2021 Jul 7;11:13665. doi: 10.1038/s41598-021-93006-0 (PMC8263781; doi:10.1038/s41598-021-93006-0)
Supplement: Supplementary file 1 — Supplementary Information. [file 41598_2021_93006_MOESM1_ESM.pdf]

Supplementary Information

Recent decadal enhancement of Meiyu–Baiu heavy rainfall over East Asia

Hiroshi G. Takahashi [1*] and Hatsuki Fujinami [2]

Extended Data Figure Legends

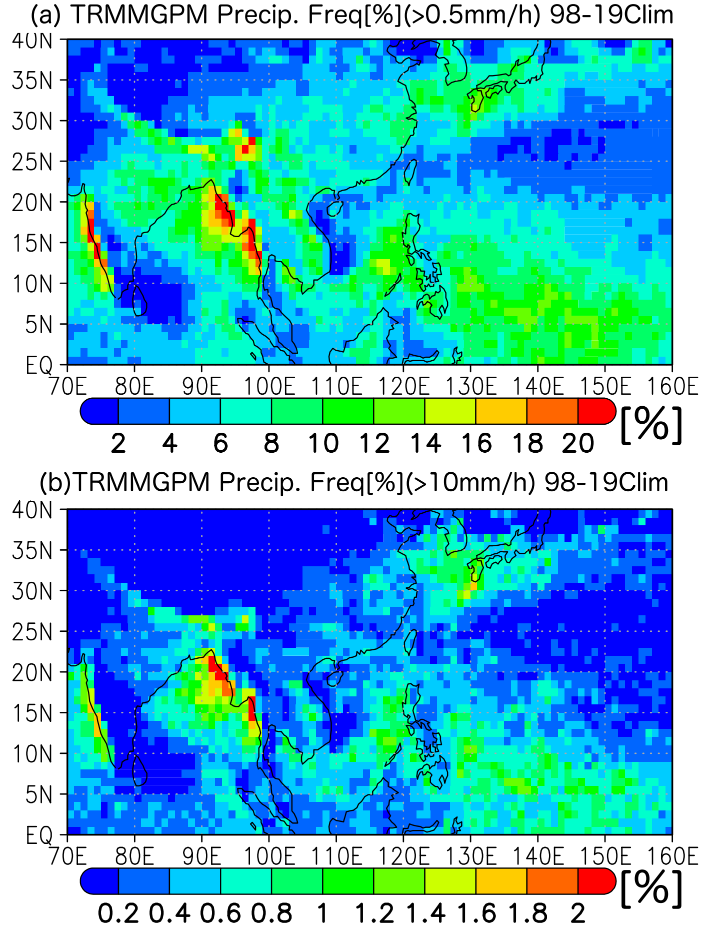

Extended Data Figure 1| (a) Frequency of Meiyu–Baiu rainfall. Precipitation events were counted when the rainfall rate exceeded the threshold value. The threshold value was 1.0 mm/h in **(a)** and 10.0 mm/h in **(b)**. Climatological frequency for Fig. 2. The rainfall

frequency, expressed in percent, was defined by dividing the number of the observed surface precipitation pixels by the total number of observation pixels. For plotting, the rainfall frequency was compiled on a 1° × 1° grid. Figure generated with GrADS 2.2.1 (http://cola.gmu.edu/grads).

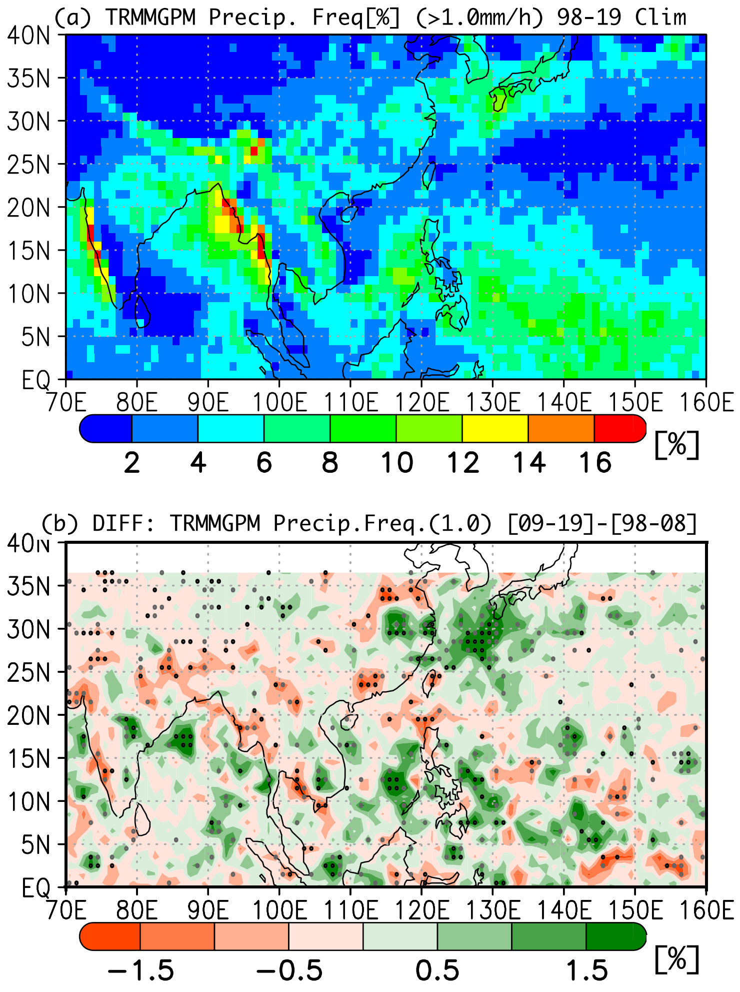

Extended Data Figure 2| (a) Frequency of Meiyu–Baiu rainfall and **(b)** its 22-year changes with confidence intervals indicated by black (95%) and grey (90%) dots. Precipitation events were counted when the rainfall rate exceeded the threshold value. The

threshold value was 1.0 mm/h. The rainfall frequency, expressed in percent, was defined by dividing the number of the observed surface precipitation pixels by the total number of observation pixels. For plotting, the rainfall frequency was compiled on a 1° × 1° grid. No data is indicated by not plotting basically north of 36° N. During the TRMM-era (1998–2013), only tropical and subtropical regions were observed due to the satellite orbit of the TRMM. Figure generated with GrADS 2.2.1 (http://cola.gmu.edu/grads).

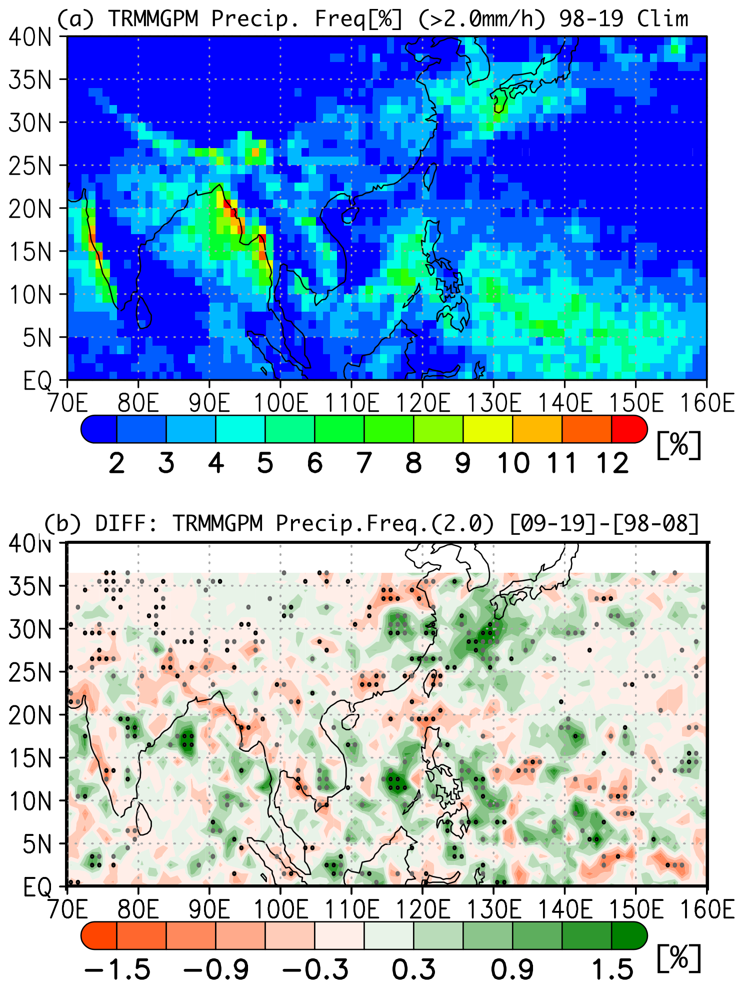

Extended Data Figure 3| As shown in Extended Data Fig. 2 but with a threshold value of

2.0 mm/h. Figure generated with GrADS 2.2.1 (http://cola.gmu.edu/grads).

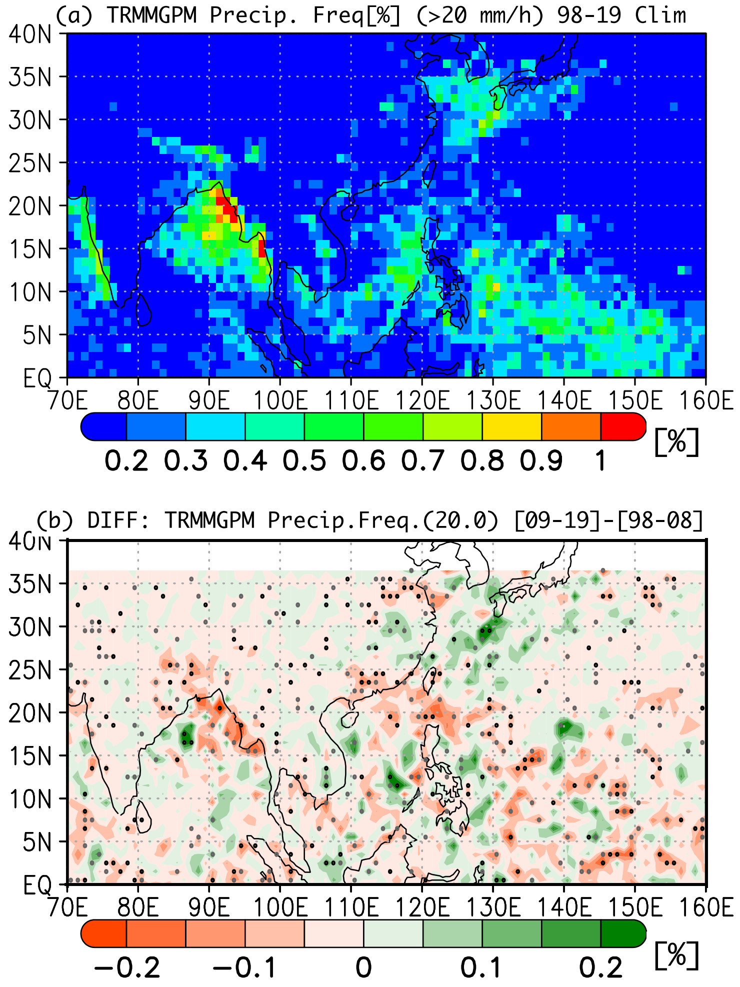

Extended Data Figure 4| As shown in Extended Data Fig. 2 but with a threshold value of 20.0 mm/h. Figure generated with GrADS 2.2.1 (http://cola.gmu.edu/grads).

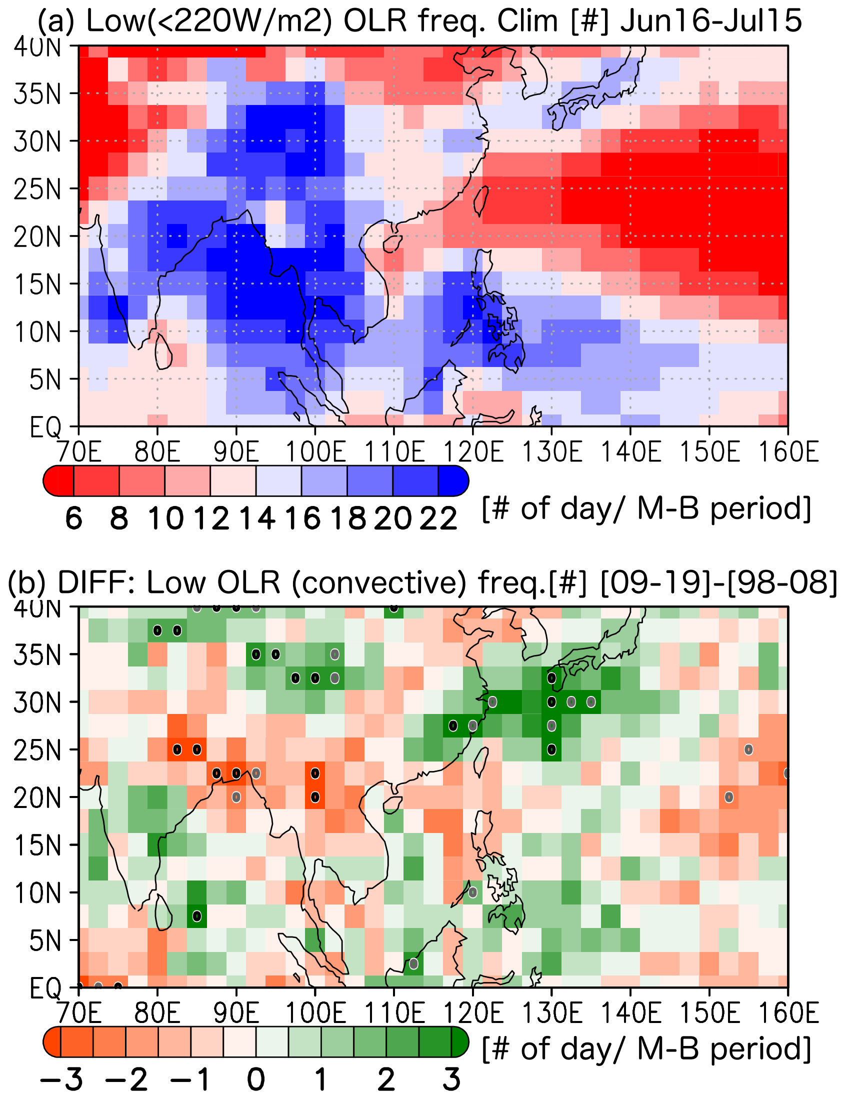

Extended Data Figure 5| As shown in Extended Data Fig. 2 but with the frequency of convective activity derived from OLR data. Figure generated with GrADS 2.2.1 (http://cola.gmu.edu/grads).

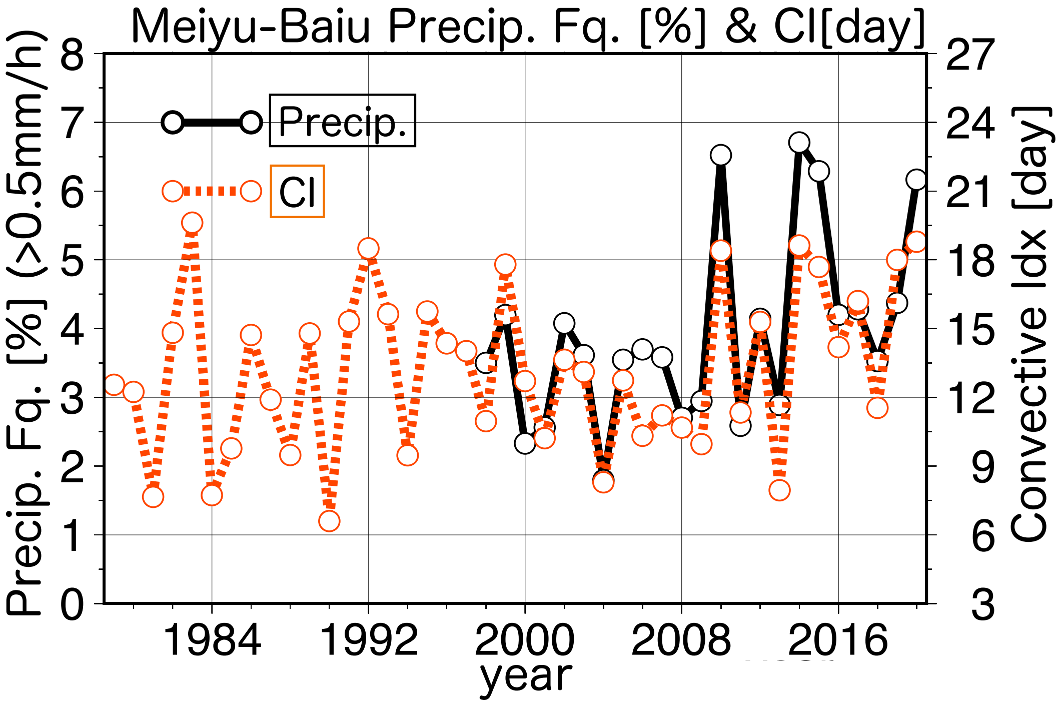

Extended Data Figure 6| Time series of interannual variations in the frequency of Meiyu–Baiu band precipitation (27–33º N, 115–135º E). Precipitation (0.5 mm/h; black solid line with closed circles) and developed clouds (220 W/m^2; red dashed line), frequencies observed by precipitation radar from 1998–2020 and derived from OLR between 1979 and 2020, respectively. The definition of rainfall (developed clouds) frequency is the same as in Extended Data Fig. 1 (Extended Data Fig. 4). The frequencies were averaged along the Meiyu–Baiu band in the Meiyu–Baiu season of each year.

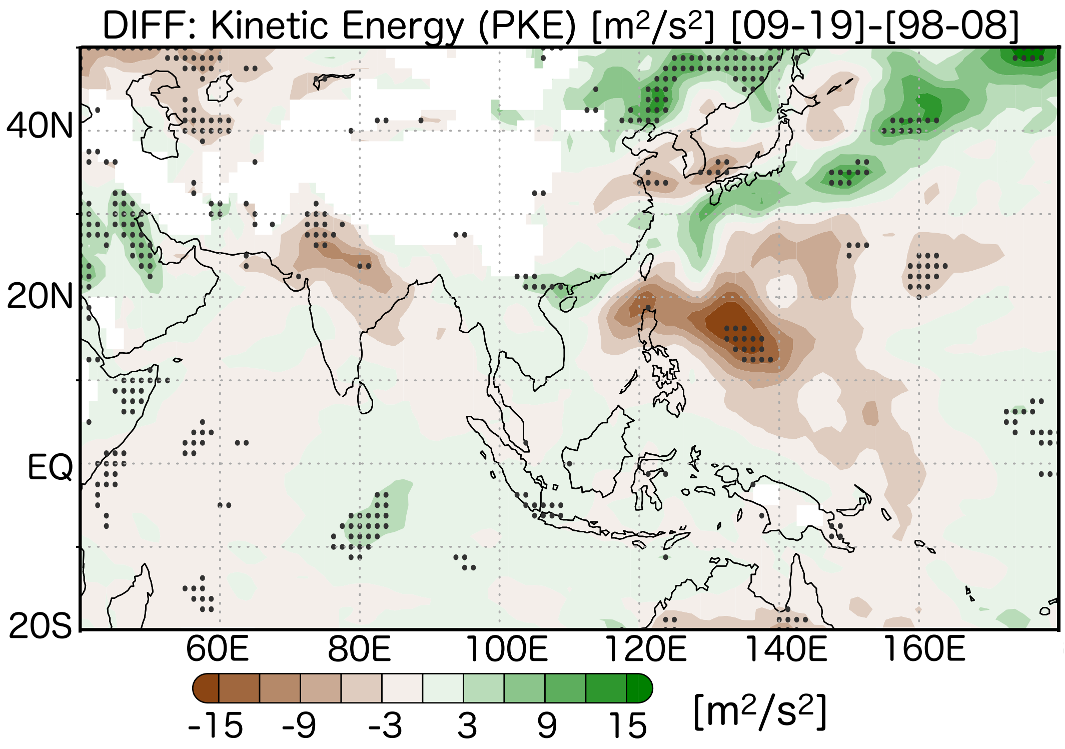

Extended Data Figure 7| As shown in Fig. 4(a) but with 850-hPa PKE. The statistical significances at a 90% confidence interval are shown by black dots. Figure generated with GrADS 2.2.1 (http://cola.gmu.edu/grads).

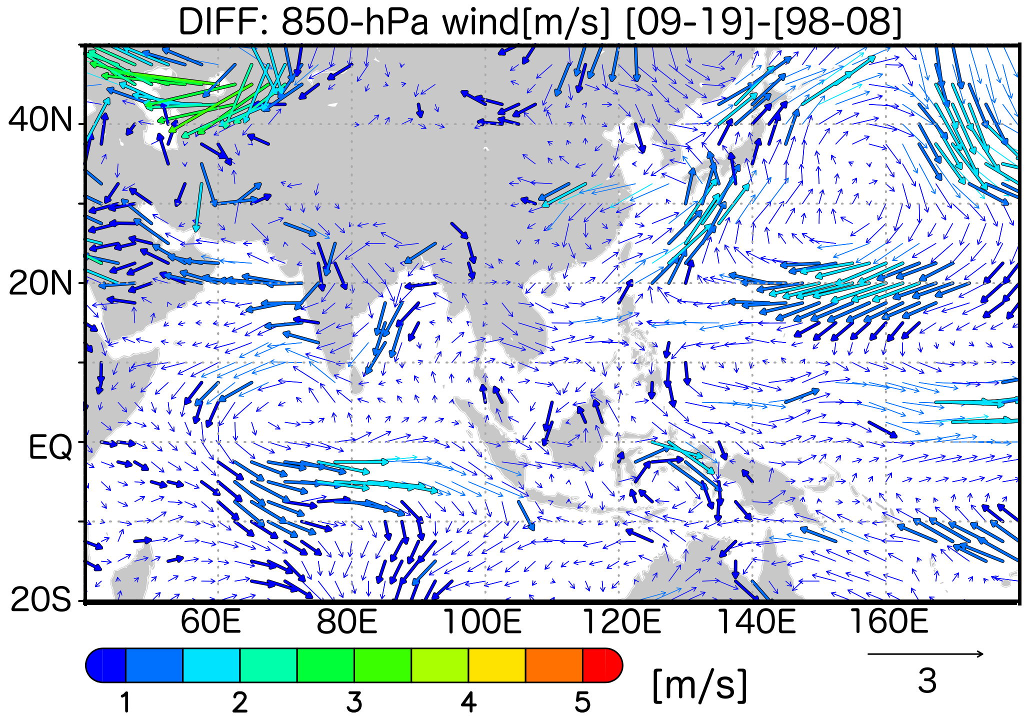

Extended Data Figure 8| As shown in Fig. 4(a) but with 850-hPa winds. The statistical significances at a 95% confidence interval are indicated by the black lines drawn around the edges of the vectors. Figure generated with GrADS 2.2.1 (http://cola.gmu.edu/grads).

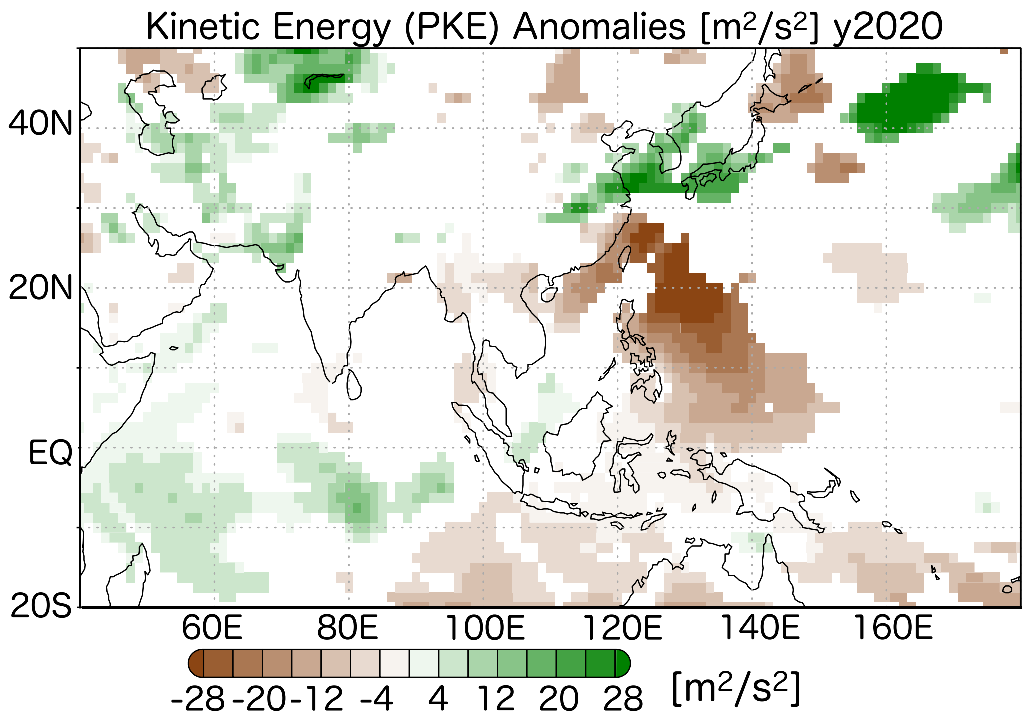

Extended Data Figure 9| As shown in Fig. 1(b) but with 850-hPa PKE. Anomalies were deviations from the climatological mean from 1998–2019. When an absolute value of an anomaly was less than the year-to-year standard deviation, it was not plotted. Figure generated with GrADS 2.2.1 (http://cola.gmu.edu/grads).

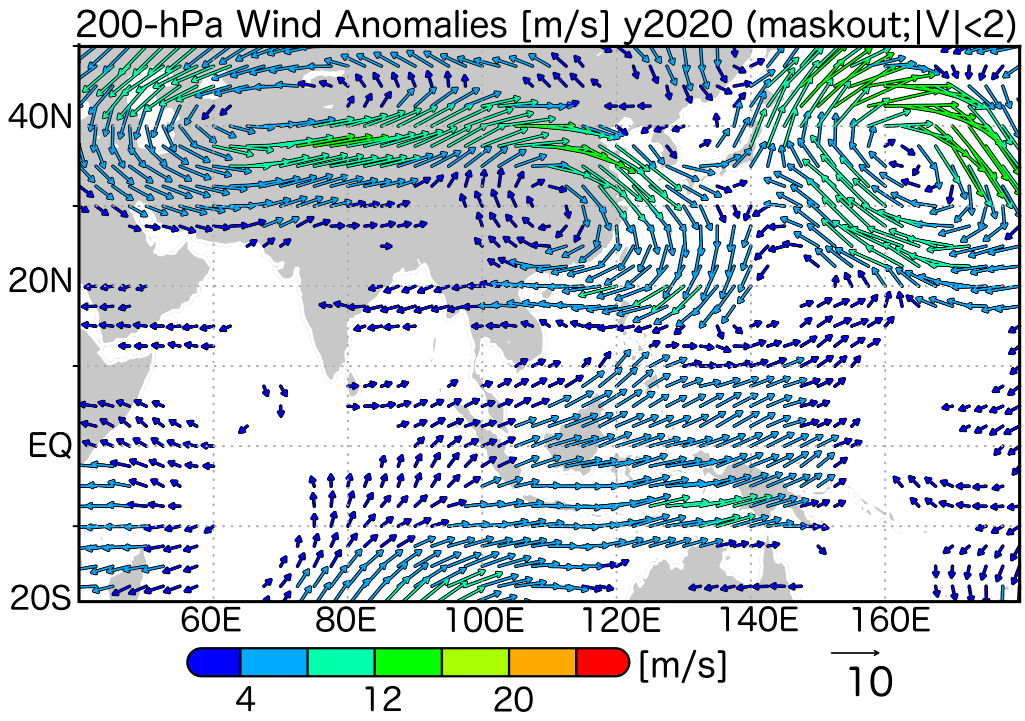

Extended Data Figure 10| As shown in Fig. 1(b) but with 200-hPa winds in the 2020 Meiyu–Baiu season. Vector colours denote their magnitudes. Anomalies were deviations from the climatological mean from 1998–2019. When the absolute value of an anomaly was less than 2 m/s, it was not plotted. Figure generated with GrADS 2.2.1 (http://cola.gmu.edu/grads).

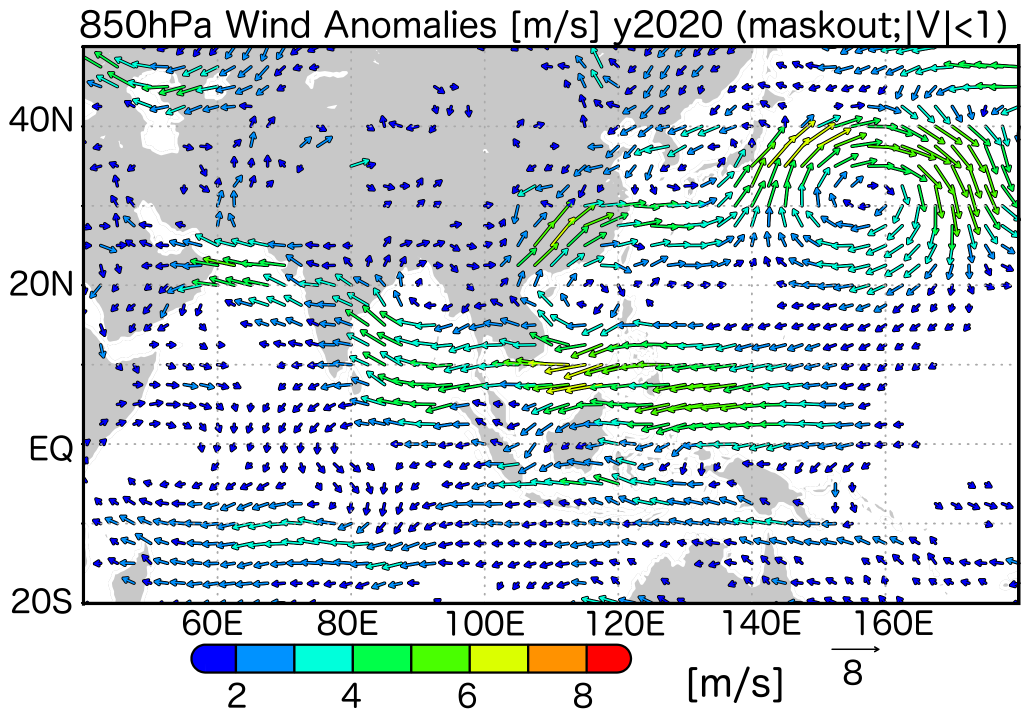

Extended Data Figure 11| As shown in Fig. 1(b) but with 850-hPa winds in the 2020 Meiyu–Baiu season. Vector colours denote their magnitudes. Anomalies were deviations from the climatological mean from 1998–2019. When the absolute value of an anomaly was less than 1 m/s, it was not plotted. Figure generated with GrADS 2.2.1 (http://cola.gmu.edu/grads).
